# Practical Guidance for Studies Using Freelisting Interviews

**DOI:** 10.5888/pcd17.200355

**Published:** 2021-01-14

**Authors:** Shimrit Keddem, Frances K. Barg, Rosemary Frasso

**Affiliations:** 1 Michael J. Crescenz VA Medical Center, Philadelphia, Pennsylvania; 2 University of Pennsylvania, Perelman School of Medicine, Philadelphia, Pennsylvania; 3 Jefferson College of Population Health, Thomas Jefferson University, Philadelphia, Pennsylvania

## Abstract

Freelisting is a qualitative interviewing technique that has recently grown in popularity. It is an excellent tool for rapidly exploring how groups of people think about and define a particular health-related domain and is well suited for engaging communities and identifying shared priorities. In this article, we outline 7 practical considerations for conducting freelisting studies summarized from 16 articles conducted by the authors at the University of Pennsylvania and Thomas Jefferson University in partnership with community-based organizations and students. Our recommendations can inform study design, data collection, and data analysis for investigators who are interested in using freelisting interviews in their research.

**Summary**

**What is known on this topic?**

Freelisting is a qualitative interviewing technique used for exploring how groups of people think about a health-related topic in order to engage the community.

**What is added by this report?**

We outline 7 practical considerations for conducting freelisting.

**What are the implications for public health practice?**

Our recommendations can help inform study design and data collection and analysis for researchers who are interested in using freelisting.

## Introduction

Freelisting, a qualitative method developed by anthropologists in the early 1980s (1,2), can be used as an elicitation technique during one-on-one interviews or in group settings to define a particular domain. Researchers start by asking participants to name all the items that come to mind in response to a prompt (2) (eg, “What comes to mind when you think about staying healthy while in school?”). Items are sorted and ranked by the researcher on the basis of frequency or salience, a measure indicating the importance of an item to the respondents. The data allow researchers to understand how a population defines various health-related topics, from perceptions of illness to health behavior to health care needs. The strength of this method is that it elicits unimagined, spontaneous responses that can be rapidly collected, analyzed, and quantified, and results from these analyses can be incorporated into mixed-methods studies in different populations and settings. The information gained from freelisting interviews can be invaluable to understanding community needs and priorities and informing culturally appropriate health communication, health promotion, and research materials. The terms “freelisting,” “free listing,” and “free-listing” are often used interchangeably. A Web of Science search including these terms returned only 2 articles during 1999; by 2019, the cumulative number of articles using freelisting had increased to 361.

The increasing use of qualitative and mixed-methods approaches has led to the growing application of freelisting in health research, especially among vulnerable populations such as people living in low-income urban settings, people who inject drugs, and people with serious mental illness. Despite this growth, few published resources detail the intricacies of designing and implementing freelisting studies. The lack of resources providing guidance is a barrier to researchers conducting thoughtful and rigorous studies that incorporate freelisting either on its own or in combination with other methods.

We analyzed a convenience sample of 16 studies (3–18) conducted by the authors at 2 Philadelphia institutions (the University of Pennsylvania and Thomas Jefferson University) in partnership with community-based organizations and students (Table). Our goal was to summarize the methodologic information in the studies to provide practical guidance for conducting freelisting interviews. The 16 studies include varying health topics in differing populations, with both freelisting used on its own or as part of mixed-methods studies or community-based participatory work. We outline the potential challenges and considerations in conducting freelisting interviews and suggest practical solutions, including tactics for designing a freelisting study; collecting, managing, and analyzing freelisting data; and developing the interview guide. As with all other qualitative methods, freelisting studies should incorporate rigorous systematic and reflective processes that ensure both internal and external validity and limit bias (19). Although complete objectivity is unachievable in research, researchers should strive for “strong” objectivity, whereby the researcher and their team are reflective and transparent throughout the research process (20).

**Table:** Examples of Studies That Used Freelisting

Article Authors	Population	Sample Size	Health Topic	Mixed Methods	Purpose/Final Products	Consensus Analysis	Types of Comparisons	Interview Setting or Format
Ahmad et al (3)	Heart failure patients, caregivers, clinicians	157	Hospital readmission among heart failure patients	Community-based participatory research	Inform heart failure management strategies to reduce hospital readmission	No	Clinician vs caregiver vs patient	In-person or on the telephone
Auriemma et al (18)	Intensive care unit patients and related family members	45	Intensive care unit	None	Understand patient and family priorities for critical care	Yes	Patients vs family members; family members of patients who survived vs died	In-person, collected on audio and professionally transcribed
Barg et al (4)	Adolescents or high school students	193	Injury prevention/driving safety	Focus groups	Inform messages about driver safety	No	“Good” vs “safe” driver; gender; race/ethnicity	In-person during focus groups
Bennett et al (5)	Pregnant women in low-income urban areas	40	Literacy and use of maternal health care resources	Concurrent mixed-method design; chart abstraction; literacy assessment; confirmatory focus groups	Inform patient–provider communication in prenatal care	Yes	Literacy level	In-person during study enrollment interview
Cunningham et al (6)	Master of public health students	360	Public health pedagogy/student health	Community-based participatory research	Expose students to qualitative methods and create infographics informed by community priorities	No	None	In-person intercept on campus
Dress et al (7)	Adults with psychosis, caregivers of adults with psychosis, and clinicians who care for adults with psychosis	65	Early psychosis	None	To inform care for patients with early psychosis	No	Patients vs caregivers vs clinicians	In-person
Fiks et al (8)	Pediatricians and parents of children with ADHD	90	ADHD	None	Inform shared decision making among pediatricians and parents of children with ADHD	Yes	“ADHD” vs “mental health”; parents vs clinicians; race	
Harris et al (9)	People who inject drugs and health care providers	62	Harm reduction/supervised injection facilities	Sequential mixed methods design; in-depth interviews	To inform US policy about harm reduction interventions for people who inject drugs	No	None	In-person at syringe exchange programs
Hwang et al (17)	Physicians, medical students, and nursing students	91	Clinicians’ knowledge about e-cigarettes	Survey	To inform curriculum development for medical school, nursing school, and residency program	No	Attending physicians vs residents vs medical students vs nursing students	Students approached on campus; physicians invited by email and interviewed in-person
Jonas et al (10)	Pediatricians	207	Perceptions of cost in health care	None	To inform and develop a curriculum that teaches about costs and value in pediatrics	No	Years in practice; clinical time; division/specialty	Online survey link
Karlawish et al (11)	Adult Latino and non-Latino caregivers and non-caregivers	120	Alzheimer disease	None	To inform culturally appropriate Alzheimer disease communication materials	Yes	Ethnicity; caregiver vs non-caregiver	In-person at home; vignettes
Keddem et al (12)	Adults in low-income areas who have asthma	35	Asthma control	Sequential mixed-methods design; geographic information systems; in-depth interviews; community-based participatory research	To inform and create a map of perceived geographic “hotspots” of poor asthma control	No	Zip code; sex; race; BMI; controlled vs uncontrolled asthma	In-person at home
Lucan et al (13)	African American adults	40	Healthy eating	Community-based participatory research	To inform interventions that promote healthy food consumption among African Americans in low-income areas	No	Gender	In-person; visual cues
Meghani et al (14)	Adult patients with cancer	65	Opioid self-management	Sequential multimethod; semi-structured interviews	To investigate cancer patients’ reports of opioid self-management practices and concerns to inform policy and practice interventions	Yes	None	In-person in the waiting area or with an online survey
Mollen et al (15)	Adolescent females in urban areas	30	Pregnancy, contraception, and emergency contraception	In-depth interviews	To gain insight into adolescents’ understanding of pregnancy prevention and inform provider–patient communication	No	Age; history of sexual activity	In-person in private office space in the hospital
Takeshita et al (16)	Adults with moderate to severe psoriasis	68	Disparities in psoriasis treatment	In-depth interviews	To understand racial differences in perceptions of psoriasis treatment and help explain disparities in treatment	No	Psoriasis therapies; race, education, income	NA

Abbreviations: ADHD, attention deficit hyperactivity disorder; BMI, body mass index; NA, not applicable.

## Guidelines for Conducting Freelisting Interviews

### Design the freelisting study to examine specific populations’ perspectives and compare across domains or groups

Freelisting rapidly explores the “emic,” or insider, perspective of a group or culture by asking members of that group to list all the elements of a particular domain. Central to this approach is the notion that shared cultural beliefs tend to produce shared concepts among members of that group. As a rule of thumb, a sample of 20 freelisting interviews is adequate to reach saturation in homogeneous groups, meaning that beyond this, additional interviews will likely not yield novel data (2). Freelisting allows the researcher to conduct 2 types of comparisons: 1) between different constructs (eg, different psoriasis treatments) or 2) between different groups or cultures (Figure). For comparisons across groups, we recommend aiming for at least 20 interviews per group. Of the 16 articles we analyzed, 11 contained some type of comparison across groups or domains.

**Figure:**
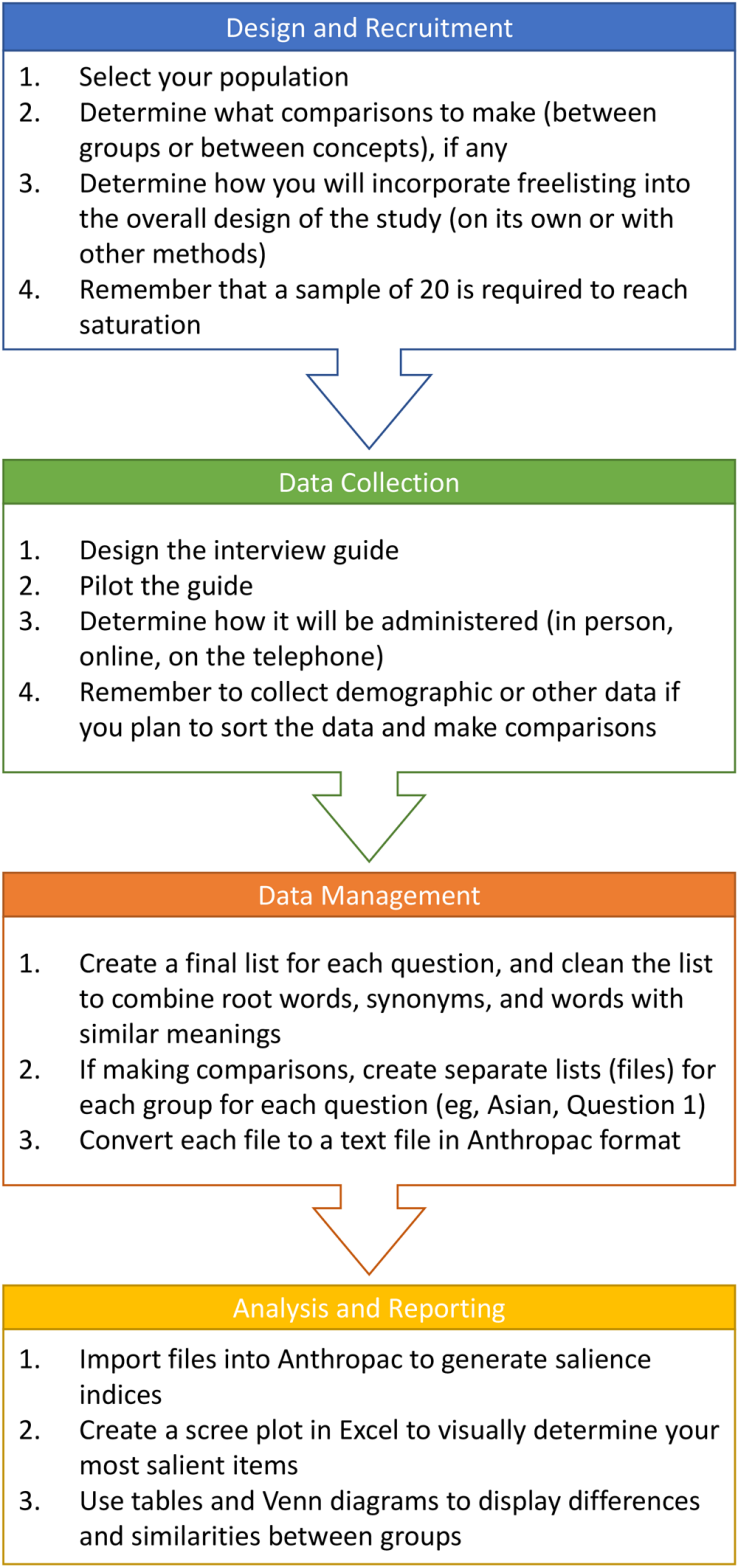
Phases of the freelisting process.

These types of comparisons can be particularly informative when researching groups with unique perspectives. For example, Barg et al (4) interviewed adolescents to understand teens’ and predrivers’ definitions of the phrases “good driver” versus “safe driver.” Each class of students was asked by a facilitator to produce 2 written lists in response to 2 prompts: “What words do you think of when you think of a good driver?” and “What words do you think of when you think of a safe driver?” Analyses aimed to compare the most salient words used for “good driver” versus “safe driver” to understand how these domains differ for young adults. In addition, the authors examined differences in responses by sex and ethnicity.

Comparisons can be used to understand linguistic and cultural differences between groups. Karlawish et al (11) compared the perceptions of 4 groups — Latinx versus non-Latinx and caregivers versus noncaregivers —of Alzheimer disease and its causes and treatments. Interviews were conducted in Spanish and English and comparisons were made across the 4 groups.

### Use freelisting in mixed-methods or qualitative studies

Freelisting can be used on its own or be incorporated into a sequential or concurrent mixed-methods design. At the beginning of any study, freelisting interviews can provide the definition and boundaries of the topic being studied (2) and therefore help develop recruitment materials, surveys, or interview guides. Freelisting findings can also aid in the development of educational materials or help determine priorities for policy or practice.

A freelisting exercise can be the first step in a study to develop an interview guide. To better understand issues surrounding supervised injection facilities, Harris et al (9) conducted freelisting interviews with people who inject drugs and health care providers recruited from a syringe exchange program. Findings were combined with policy/makers’ priorities to inform the development of a semi-structured interview guide to explore whether people who inject drugs believe that safe injection facilities could improve safety and mitigate risk of infection.

Keddem et al (12) conducted a freelisting study as part of a sequential mixed-methods design. Freelisting questions were included at the beginning of an in-depth interview guide to understand neighborhood and environmental barriers to asthma control. Freelists were collected from a stratified sample of adults with asthma living in an urban neighborhood. Freelist responses, sorted by salience, were used to generate a composite map of geographic areas of poor asthma control, as perceived by residents of the neighborhood.

Freelisting is an excellent method to use for working with communities. Minimal training is required to conduct the freelisting interviews, and the process of cleaning and analysis is enhanced by community member participation. In their freelisting study of fruit, vegetable, and fast food consumption, Lucan and colleagues (13) asked selected community members to review and help revise study documents, provide space to conduct interviews, and advertise the study to the community. Community stakeholder perspectives can also be incorporated into the data collection, management, and analysis phases. For example, Ahmad et al (3) included a patient representative with heart failure in the data cleaning and review process of a study that examined heart failure management and hospital readmissions.

Freelist interviews can take as few as 5 minutes to complete, depending on the number of questions (6). Interviews can be scheduled in advance or conducted on the spot in community settings, waiting rooms, or other places where people can be approached and can provide consent (21). Freelisting is an efficient way to gather data from large samples of people in a short time (6). Freelisting can be used when issues are time sensitive or if a group happens to be gathered together (eg, at a convention or community event) to quickly obtain and understand community goals and needs. This feature could prove useful in the context of disaster response.

### Create a freelisting interview guide

Designing the interview guide is an integral component of the freelisting study but asking the “right” questions is not necessarily easy, so piloting the guide is important. As with all qualitative research endeavors, the interview guide is strongest when reviewed by an interdisciplinary team and, if possible, with input from the community. For example, in the study by Dress et al (7) of people with early psychosis, the guide was reviewed by 4 faculty members and piloted during a focus group consisting of research faculty and staff who were familiar with the study population.

Questions should aim to elicit as extensive a list of items as possible. When respondents’ lists are too short, this can be an indication that questions are not well conceived (22). To capture the respondents’ own words, responses should be recorded verbatim by the interviewer. Because people are not accustomed to providing answers in the form of a list, starting the interview with a practice question (eg, “If I asked you to list all the names of animals that you can think of, what would you say?”) can be beneficial. The interviewer should encourage 1-word answers, but more descriptive phrases are also acceptable. It is important and appropriate for the interviewer to clarify responses that are unclear after the participant completes their list.

Data collection can be conducted in various formats and settings. Lists may be collected by the interviewer in person or on the telephone (3) or provided by the respondents, written by hand or through an electronic survey (10). Allowing interviewees an unlimited number of responses is ideal.

Eliciting listed responses to vignettes or visual cues is also possible. In the study by Lucan et al, interviewees were asked to provide a list in response to images of different foods to understand their perceptions of the food (13). Karlawish et al (11) incorporated vignettes about Alzheimer disease into their interview guide to understand the dynamics in relationships where one spouse is a caregiver for a partner who has Alzheimer disease. All caregivers were also asked to provide a list in response to a 3-paragraph description of a clinical trial for persons with Alzheimer disease.

### Develop a systematic approach for cleaning the freelisting data

Freelisting data must be carefully cleaned before analysis by reviewing raw lists to combine root words, synonyms, and similar concepts, a process that can be done in multiple iterations. On a first round, researchers, and when possible community members, should group grammatical forms of the same word (eg, “smell” and “smells”). On a second round, synonyms can be combined (eg, “scent” and “smells”), and on a third round, words representing similar concepts can be grouped (eg, “perfume,” “deodorant,” “scent,” and “smell”). Categories should be created inductively without any a priori set of rules. At this phase of the process, reviewers should be blinded to any demographics to reduce bias. To ensure the integrity of the process, it can be constructive for team members to check each other. For example, to reduce bias, the Dress et al team had 2 researchers combine synonyms, and a third researcher review the agreed-upon categories to produce the final list. If lists are collected in different languages (as with Karlawish et al), creating 2 independent translations of the lists and reviewing both to generate a final English translation for analysis is ideal.

### Use a scree plot and design a meaningful review process to interpret salience indices

The goal of the freelisting analysis is threefold: 1) to identify the most salient items across multiple respondents’ lists; 2) to present only the terms on the list that the researcher believes are truly shared by the group or groups; and 3) to develop a manageable list that is not too long (23). Anthropac software (Analytic Technologies) is a free program (www.analytictech.com/anthropac/anthropac.htm) created for the analysis of freelisting data. Freelists can be stored in many spreadsheet formats but need to be converted to a text file before they are entered into Anthropac. If multiple groups or questions are included in the study, a separate file should be generated for each question and each group within each question before importing into the software.

Smith’s S, the salience index, is the main statistic used for analyzing freelists (24). This statistic considers both the frequency of an item across all respondents’ lists as well as its rank order on these lists, reflecting the assumption that items mentioned early and often across respondents are the most salient. The salience index is a gross mean percentile rank for each item across all lists, as represented in the following equation (24):

$Freelist\;salience\;of\;an\;item\;\left(Smith’s\;S\right)=\frac{sum\;of\;the\;item’s\;percentile\;ranks}{total\;number\;of\;lists}=\frac{\sum \left(\frac{L-R_{j}}{L}\right)}{N}$

Freelist salience of an item (Smith’s S) equals the sum of the item’s percentile ranks divided by the total number of lists, where *L* is the length of each list, *R_j_* is the rank of item *j* in the list, and *N* is the number of lists in the sample.

Anthropac combines all respondents’ lists into a composite list and provides a salience index for every item on the composite list. Selecting items to display in a final report or article may be determined by many factors, including the purpose of the study, the number and frequency of the items, the number of questions asked, and the study design and format. Examining salience indices using a scree plot (in Excel [Microsoft Corporation] or other program), placing the salience index on the y axis and the items on the x axis, can help in the selection process (4,14). This setup will display the results of each question as a downward curve on a graph. There is almost always an “elbow” in the curve, which is the point where data level off. This point can be used as a cutoff, and the researcher can choose to display only items that appear before the cutoff as the most salient terms. In some cases, researchers may choose their own cutoff, for example, including only the top 5 items on each list.

Researchers can display differences between groups in several ways. Visuals and diagrams can be particularly helpful for highlighting differences. Of the 16 articles we included, 6 used Venn diagrams and 9 used tables to display differences between groups. Jonas et al (10) chose to transform the salience index into a relative salience score for comparing across groups by dividing the raw salience indices for each term by the raw index for the most salient term in the list, then converting to a percentage. In their article, they displayed these differences in tables with color coding to show differences in relative salience.

### Build on your study by adding cultural consensus analysis

Beyond the salience index, the researcher can also use consensus analysis to determine the degree of similarity among respondents. Consensus analysis is based on the theory that members of the same cultural group will have more knowledge about a given cultural domain (19). The cultural consensus analysis model relies on 3 assumptions: 1) that there is a “common truth,” or one unifying cultural reality; 2) that respondents are independent of each other, and that if they don’t know the “culturally appropriate answer,” they make one up independently; and 3) that all questions are on the same topic (25). Cultural consensus analysis generates eigenvalues, used to measure agreement among persons in a group and indicating the strength of the consensus. If there is 1 culture, there must be 1 large eigenvalue. Therefore, results are analyzed in terms of the ratio of the first eigenvalue to the second eigenvalue. If the ratio is less than 3 to 1, the researcher can assume that there is more than 1 “correct” cultural definition within the group (25). Of the 16 articles we included, 3 presented results from cultural consensus analysis.

Fiks and colleagues (8) conducted a freelisting study to understand perception of attention deficit hyperactivity disorder (ADHD) among pediatricians and parents of affected children. They included cultural consensus analysis to determine the degree to which subgroups of parents and clinicians shared an understanding of ADHD. For each group and for each question, they used Anthropac to measure how each person’s response was weighted in relation to the most frequent responses of the group. They looked for an eigenvalue greater than 3 to indicate group consensus and found that none existed among clinicians for any of the freelist questions. However, parental consensus was found for 2 questions: 1) words participants think about when they hear “ADHD” and 2) getting help for ADHD. They also assessed consensus by race and found no strong consensus among White participants to any of the questions. Among Black participants, consensus was found for items describing ADHD.

### Understand the limitations of freelisting

Freelisting interviews have several limitations. Freelisting responses consist only of individual words or phrases and do not provide the type of depth that can be gained from a semi-structured or extensive qualitative interview or focus group. However, freelisting elicits a set of spontaneous responses in the respondents’ own words. To overcome this barrier, researchers can and should incorporate freelisting in mixed-methods studies as a step in the research process to provide triangulation and inform subsequent steps of the process. In the study by Bennett et al (5) of the role of low literacy in maternal care utilization, the researchers conducted confirmatory focus groups following freelisting to confirm and explore the items identified by respondents. Another limitation is the potential bias introduced during data cleaning. To reduce this bias, it is important that the cleaned categories be inductive and use the respondents’ words. In addition, developing a rigorous and systematic cleaning process is also necessary, ideally one involving multiple perspectives from team and community members.

## Conclusion

This article outlines guidelines and considerations for conducting freelisting studies summarized from 16 articles conducted by researchers at the University of Pennsylvania and Thomas Jefferson University. Our findings can be useful to investigators interested in conducting freelisting studies by providing practical and methodologic considerations that may arise at the design, data collection, and analysis stages of the research.

Freelisting is an ideal technique for understanding the community perspective and eliciting spontaneous responses that can allow researchers to gain insight into the insider perceptions of a variety of health topics. Results from freelisting interviews can be gained rapidly, are quantifiable, and can be immediately applied in research and practice. Researchers who are interested in using freelisting should consider their population of interest, identify the purpose of their study, and understand the theoretical and methodological underpinnings of the technique.
